# Reply to Krefeld-Schwalb et al.: Measuring population heterogeneity requires upholding good scientific practice

**DOI:** 10.1073/pnas.2426330122

**Published:** 2025-02-18

**Authors:** Felix Holzmeister, Magnus Johannesson, Robert Böhm, Anna Dreber, Jürgen Huber, Michael Kirchler

**Affiliations:** ^a^Department of Economics, University of Innsbruck, Innsbruck A-6020, Austria; ^b^Department of Economics, Stockholm School of Economics, Stockholm SE-11383, Sweden; ^c^Department of Occupational, Economic, and Social Psychology, University of Vienna, Vienna A-1010, Austria; ^d^Department of Psychology and Center for Social Data Science, University of Copenhagen, Copenhagen DK-1353, Denmark; ^e^Department of Banking and Finance, University of Innsbruck, Innsbruck A-6020, Austria

We appreciate Krefeld-Schwalb et al.’s (KHJ henceforth) (1) interest in our study (2) and critical discourse spurred by their commentary. We largely agree with KHJ’s theoretical arguments, which closely relate to the caveats discussed in our manuscript. Particularly, we acknowledge that the reviewed multilab studies are typically based on samples from WEIRD countries, which may entail lower heterogeneity than in other settings (3, 4). “Put differently, our comparatively low estimates of population heterogeneity might be subject to population heterogeneity itself.” (2).

However, we express reservations about KHJ’s empirical claims about the magnitude of population heterogeneity, drawing on Krefeld-Schwalb et al. (KSJ henceforth) (5). It seems that KSJ intentionally studied paradigms expected to yield large meta-analytic effect sizes and “employed purposive variation of the sampling frame” (1) to enhance heterogeneity. Olsson-Collentine et al. (6) provide evidence for a correlation between effect sizes and heterogeneity. Study 1 in KSJ documents effect size estimates of four paradigms across ten online samples and one laboratory sample. KHJ’s estimates of *H*, ranging from 1.7 to 9.6, suggest that population heterogeneity is markedly larger than the average level of population heterogeneity observed in our sample. However, both KSJ and KHJ fail to report estimates for a fifth preregistered paradigm embedded in KSJ’s study—the “local warming” effect—and omit preregistered analyses excluding inattentive participants. Table 1 summarizes heterogeneity estimates for analyses mimicking KSJ’s preregistration. Revisiting KSJ’s data on the local warming effect indicates that effect size estimates are homogeneous with *H* = 1. Moreover, population heterogeneity estimates for the five paradigms turn out to be lower after excluding inattentive participants. KHJ also reports heterogeneity estimates for studies 2 and 3 in KSJ, which are, however, based on only two samples each. Quantifying heterogeneity based on very small numbers of studies (*k*) has been shown to be inappropriate and can be misleading; for *k* = 2, *H* is uninformative since *H* = *Q* ÷ (*k*−1) = *Q*. (7) After all, heterogeneity in KSJ appears to be much smaller than suggested by KHJ, with *H* estimates ranging from 1.0 to 3.2 when including inattentive subjects and between 1.0 and 1.6 when excluding inattentive subjects.

**Table 1.** Heterogeneity estimates based on KSJ’s data

	All samples (*k* = 11)		Online marketplaces (*k* = 10)	
Paradigm (measure)	Incl. inattentive participants^†^ (KHJ’s estimate)	Excl. inattentive participants^‡^	Incl. inattentive participants^†^	Excl. inattentive participants^‡^
Default effect (Cohen’s *h*)	*Q*(10) = 50.4 *P* < 0.001 *H* = 2.3 [1.6, 4.0] *I*^2^ = 80.8 [60.3, 93.8]	*Q*(10) = 20.0 *P* = 0.029 *H* = 1.4 [1.0, 2.8] *I*^2^ = 49.1 [0.0, 87.1]	*Q*(9) = 41.2 *P* < 0.001 *H* = 2.2 [1.5, 4.0] *I*^2^ = 78.7 [54.7, 93.7]	*Q*(9) = 18.2 *P* = 0.033 *H* = 1.4 [1.0, 2.9] *I*^2^ = 49.6 [0.0, 88.2]
Framing effect (Cohen’s *h*)	*Q*(10) = 81.2 *P* < 0.001 *H* = 3.0 [2.1, 5.4] *I*^2^ = 89.1 [77.5, 96.6]	*Q*(10) = 25.0 *P* = 0.005 *H* = 1.6 [1.1, 3.1] *I*^2^ = 62.6 [20.8, 89.4]	*Q*(9) = 80.9 *P* < 0.001 *H* = 3.2 [2.2, 5.9] *I*^2^ = 90.1 [79.0, 97.1]	*Q*(9) = 21.1 *P* = 0.012 *H* = 1.6 [1.1, 3.2] *I*^2^ = 59.8 [12.3, 90.2]
Less is better (Cohen’s *d*)	*Q*(10) = 88.3 *P* < 0.001 *H* = 3.0 [2.1, 5.4] *I*^2^ = 89.2 [77.8, 96.5]	*Q*(10) = 22.0 *P* = 0.015 *H* = 1.5 [1.0, 2.9] *I*^2^ = 55.1 [7.7, 87.9]	*Q*(9) = 88.1 *P* < 0.001 *H* = 3.2 [2.2, 5.9] *I*^2^ = 90.2 [79.3, 97.1]	*Q*(9) = 19.2 *P* = 0.024 *H* = 1.5 [1.0, 3.0] *I*^2^ = 52.8 [0.6, 89.2]
Sunk cost (Cohen’s *d*)	*Q*(10) = 28.6 *P* = 0.001 *H* = 1.7 [1.2, 3.1] *I*^2^ = 66.5 [31.0, 89.9]	*Q*(10) = 27.3 *P* = 0.002 *H* = 1.7 [1.2, 3.4] *I*^2^ = 66.1 [29.8, 91.1]	*Q*(9) = 28.4 *P* = 0.001 *H* = 1.8 [1.2, 3.4] *I*^2^ = 69.9 [35.7, 91.5]	*Q*(9) = 21.4 *P* = 0.011 *H* = 1.6 [1.1, 3.4] *I*^2^ = 59.3 [13.8, 91.1]
Local warming^§^ (Pearson’s *r*)	*Q*(10) = 7.5 *P* = 0.673 *H* = 1.0 [1.0, 1.5] *I*^2^ = 0.0 [0.0, 57.4]	*Q*(10) = 7.9 *P* = 0.642 *H* = 1.0 [1.0, 1.6] *I*^2^ = 0.0 [0.0, 59.1]	*Q*(9) = 7.4 *P* = 0.591 *H* = 1.0 [1.0, 1.7] *I*^2^ = 0.0 [0.0, 63.9]	*Q*(9) = 7.9 *P* = 0.548 *H* = 1.0 [1.0, 1.7] *I*^2^ = 0.0 [0.0, 66.9]

Notes: The raw data and code published alongside KSJ’s manuscript appear to be incomplete and are hardly annotated. All estimates are, thus, based on the preprocessed individual-level data contained in KSJ’s reproduction kit. KSJ’s preregistration (osf.io/4dyp2) does not mention data collection of a student sample in an offline setting and states that analyses are to be executed “both including and excluding flagged respondents (who either fail our attention check questions or are identified by Qualtrics as bots).” KSJ deviated from their preanalysis plan in collecting data for a lab sample of students and in not reporting analyses including bots. Columns marked with ^†^ include all participants who completed the experiment; columns marked with ^‡^ exclude participants failing at least one of two attention checks (as per KSJ’s preanalysis plan), reducing the overall sample from 6,438 to 4,037. However, all estimates pertain to samples after excluding bots since we failed to reproduce the data processing (due to incomplete raw data) to re-estimate effect sizes based on unfiltered samples. ^§^ KSJ do not report estimates for the preregistered “local warming” manipulation, remarking in SI that the effect is not included in the analysis “[s]ince [it] is not a randomized experiment.” Notably, the meta-analytic effect size (across all samples and including inattentive subjects) turned out to be close to zero and statistically insignificant (*r* = 0.016, 95% CI [−0.008, 0.041]; *z* = 1.29, *P* = 0.196) and effect size estimates are homogeneous, indicating a failure to replicate the claims of a meta-analysis by Sugerman et al. (8) (involving two of KSJ’s original authors). The table reports heterogeneity estimates in terms of Cochran’s *Q*(*df*)-tests, *H*, and *I*^2^ (in %) alongside their associated 95% CI (in brackets) for the five paradigms investigated in KSJ based on random effects meta-analyses using the restricted maximum likelihood estimator for *τ*. The tabulated estimates emulate KSJ’s preregistered analyses (osf.io/4dyp2) as closely as possible based on a random effects meta-analysis framework (refer to the table notes for details). Columns 2 to 3 report estimates based on all samples; columns 4 to 5 report estimates based on samples obtained via crowdsourced marketplaces (i.e., excluding the student sample). Columns 2 and 4 (3 and 5) include (exclude) inattentive participants. *H* estimates depicted in KHJ’s figure 1 correspond to the estimates in column 2.

How heterogeneous are the populations in KSJ? All online samples in Study 1 were drawn from “anglophone participants in three highly developed countries” (1) and turned out to be relatively similar in terms of demographic characteristics. When the crowdsourced marketplace is held constant across three samples (Prolific, Prolific US, and Prolific UK), effect size estimates are remarkably similar. This suggests that data collection via online platforms may exacerbate heterogeneity due to, for instance, different procedures for screening and compensating participants. This, in turn, may introduce variability in the number of bots, attrition rates, attention, experience, and comprehension—moderating factors for which KSJ provides supporting evidence. Consequently, the generalizability of empirical claims across and beyond varying online marketplaces may be lower than for studies based on laboratory or observational data. The extent of population heterogeneity is ultimately an empirical question that requires further investigation and evidence.
